# HIV envelope antibodies and TLR7 agonist partially prevent viral rebound in chronically SHIV-infected monkeys

**DOI:** 10.1371/journal.ppat.1010467

**Published:** 2022-04-22

**Authors:** Brian Moldt, Abishek Chandrashekar, Erica N. Borducchi, Joseph P. Nkolola, Heather Stephenson, Mark Nagel, Magdeleine Hung, Joshua Goldsmith, Craig S. Pace, Brian Carr, Nathan D. Thomsen, Wade S. Blair, Romas Geleziunas, Dan H. Barouch

**Affiliations:** 1 Gilead Sciences, Foster City, California, United States of America; 2 Beth Israel Deaconess Medical Center, Boston, Massachusetts, United States of America; 3 The Ragon Institute of MGH, MIT and Harvard, Cambridge, Massachusetts, United States of America; University of North Carolina at Chapel Hill, UNITED STATES

## Abstract

A key challenge for the development of a cure to HIV-1 infection is the persistent viral reservoir established during early infection. Previous studies using Toll-like receptor 7 (TLR7) agonists and broadly neutralizing antibodies (bNAbs) have shown delay or prevention of viral rebound following antiretroviral therapy (ART) discontinuation in simian-human immunodeficiency virus (SHIV)-infected rhesus macaques. In these prior studies, ART was initiated early during acute infection, which limited the size and diversity of the viral reservoir. Here we evaluated in SHIV-infected rhesus macaques that did not initiate ART until 1 year into chronic infection whether the TLR7 agonist vesatolimod in combination with the bNAb PGT121, formatted either as a human IgG1, an effector enhanced IgG1, or an anti-CD3 bispecific antibody, would delay or prevent viral rebound following ART discontinuation. We found that all 3 antibody formats in combination with vesatolimod were able to prevent viral rebound following ART discontinuation in a subset of animals. These data indicate that a TLR7 agonist combined with antibodies may be a promising strategy to achieve long-term ART-free HIV remission in humans.

## Author summary

In a rhesus macaque model for chronic HIV infection where ART was not initiated before 1 year of infection, we demonstrate that the HIV bNAb PGT121 (formatted in three immune cell engager formats) together with the TLR7 agonist vesatolimod can partially prevent viral rebound following discontinuation of ART. Importantly, in most of the animals that did not rebound following the ART discontinuation, CD8+ cell depletion did not result in viral rebound, suggesting that the lack of rebound was not dependent on CD8+ T or NK cells. To the best of our knowledge, no similar study has been performed in the nonhuman primate model with animals that started ART deep into chronic infection. This proof-of-concept study in chronically infected rhesus macaques supports that this treatment regimen may represent a strategy to achieve long-term ART-free HIV remission in humans.

## Introduction

Viral replication can be efficiently suppressed by ART in HIV-1 infected individuals with a significant reduction of morbidity and mortality. However, a viral reservoir in latently infected CD4+ T lymphocytes is formed very early during infection [1–4]. This viral reservoir represents the key challenge for the development of a cure to HIV-1 as the vast majority of HIV-1-infected individuals who discontinue ART experience viral rebound [5,6]. Multiple strategies to target the viral reservoir are being investigated and one approach is pairing immune modulators with HIV Env targeted immune cell engaging antibodies to activate and eliminate these latently infected cells [7,8].

We and others have previously demonstrated that a combination of vesatolimod (VES, previously known as GS-9620) [9]) or a related TLR7 agonist and the HIV-1 bNAbs PGT121 alone or in combination with N6-LS [10] can delay or prevent viral rebound in SHIV-infected rhesus macaques following ART discontinuation [11,12]. In these initial proof-of-concept studies, ART was initiated early during acute infection to minimize the size and diversity of the viral reservoir.

To explore whether this treatment strategy and outcome can be translated into a more clinically relevant model, we evaluated the capacity of VES and PGT121 to target the viral reservoir in SHIV-SF162P3-infected rhesus macaques who initiated ART during the chronic infection phase. To explore the mechanism of action of the antibody formats, we evaluated the bNAb PGT121 as a human IgG1, an effector enhanced IgG1, or an anti-CD3 bispecific antibody.

## Results

### Antibody study drugs

PGT121 is a clinical stage human IgG1 bNAb and has shown highly effective antiviral activity in animal models for HIV prevention, treatment and cure [10,11,13,14,15] (S1 Fig). GS-9721 is an engineered version of PGT121 with enhanced Fc-mediated effector functions and half-life mediated through higher binding affinities for activating Fcγ receptors (FcγRs) and the neonatal Fc receptor (FcRn) [16,17,18] (S1 Fig). The bispecific PGT121/anti-CD3 is generated in the Duobody platform [19,20] and contains a PGT121 Fv, an anti-CD3 Fv (for T-cell recruitment) and a rhesus Fc domain (S1 Fig). Bispecific PGT121/anti-CD3 is engineered to have reduced Fc-mediated effector functions through lower binding affinities for FcγRs and C1q. To stimulate immune tolerance [21], the bispecific PGT121/anti-CD3 antibody was also generated as an anti-CD3 Fv variant with a serine to histidine mutation at Kabat position 100d in complementarity-determining region 3 of the heavy chain to abrogate binding to CD3 (Bispecific PGT121/anti-CD3KO).

### Animal study design

**Fig 1:**
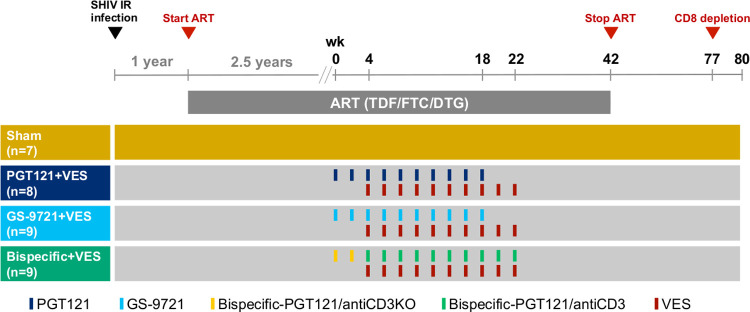
Schematic representation of study design. 33 rhesus macaques were infected interrectally with SHIV-SF162P3 and after 1 year of infection initiated on ART. Following 2.5 years of ART, animals were assigned to Sham (Group 1, n = 7), PGT121 and VES (Group 2, n = 8), GS-9721 and VES (Group 3, n = 9) or bispecific-PGT121/anti-CD3 and VES (Group 4, n = 9). Animals in Group 2 and 3 received 10 biweekly doses of antibody and VES with the first administration of VES on the day of the third antibody administration. Animals in Group 4 received first 2 biweekly doses of bispecific PGT121/anti-CD3 knock out followed by 10 biweekly doses of bispecific PGT121/anti-CD3 and VES with the first administration of VES on the day of first bispecific PGT121/anti-CD3 administration. Sham control animals received saline placebo administrations. ART was discontinuation at week 42 from initial antibody dosing and viral load was monitored. At week 77, CD8+ cell depletion was performed in a subset of animals.

Thirty-three Indian-origin rhesus macaques (Macaca mulatta) were infected intrarectally with SHIV-SF162P3 [22]. After 1 year of infection, allowing the animals to progress into the chronic infection phase, ART was initiated as previously described [23] and plasma viremia declined to undetectable levels in all animals (S2 Fig). Following 2.5 years of ART, animals were assigned to one of the following treatment groups: Sham (Group 1, n = 7), PGT121 and VES (Group 2, n = 8), GS-9721 and VES (Group 3, n = 9) or bispecific-PGT121/anti-CD3 and VES (Group 4, n = 9). Animals in group 2 and 3 received 10 biweekly doses of antibody. The first administration of VES was given on the day of the third antibody administration. Animals in Group 4 received 2 biweekly doses of CD3-inactive bispecific PGT121/anti-CD3KO followed by 10 biweekly doses of bispecific PGT121/anti-CD3. The first administration of VES was given on the day of the first bispecific PGT121/anti-CD3 administration. In single and repeat-dose studies, the bispecific PGT121/anti-CD3 molecule had previously been observed to induce extensive ADA in NHPs. Thus, 2 preliminary doses of a bispecific PGT121/anti-CD3KO were given to tolerize the animals to the bispecific antibody as previously described [21]. Sham control animals received saline placebo administrations. ART was continued for 24 weeks (20 weeks for bispecific+VES group) after the final combined antibody/VES dose to allow for antibody washout. Following ART discontinuation (week 42 from initial antibody dosing) viral rebound was monitored for 24 weeks (week 66 from initial antibody dosing). Fig 1 shows a schematic of the study design (Fig 1).

### Antibody pharmacokinetics and anti-drug antibody responses

**Fig 2:**
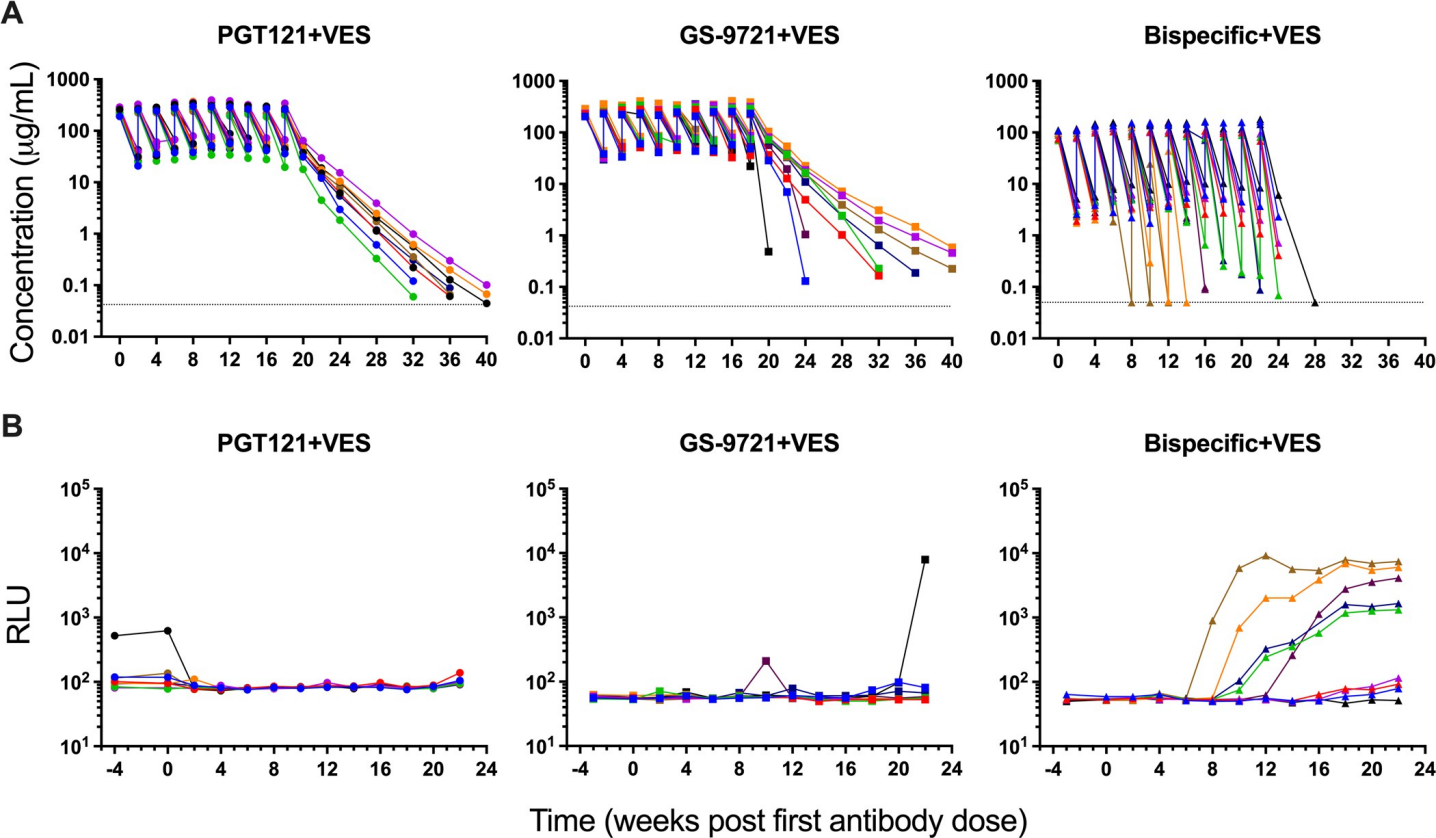
Antibody pharmacokinetics and anti-drug antibody (ADA) in serum before ART discontinuation. (A) Peak serum antibody levels are shown following each of the ten infusions of antibody and during the washout period. Dotted lines indicate limit of detection, values on the line were below limit of detection. (B) ADA responses were assessed at week -4 and every 2 weeks from week 0 to week 22. Individual symbol and color coding as indicated in S1 Table.

Antibody serum concentrations were determined following antibody dosing for animals in groups 2–4 (Fig 2A). In PGT121+VES dosed animals, antibody was detected after each of the 10 infusions with peak serum concentrations from 188 to 398 μg/mL 30 minutes post dosing. Antibody concentrations declined to between 18 and 88 μg/mL two weeks post dosing, prior to the next dose, reflecting a relatively typical half-life of the human antibody in rhesus macaques, as previously observed [13]. GS-9721+VES dosed animals reached similar antibody levels with peak serum concentrations between 205 and 412 μg/mL 30 minutes post dosing that declined to between 22 and 114 μg/mL two weeks post dosing, except for one animal where the serum concentration following the last dose declined to 0.5 μg/mL. The threshold for PGT121-mediated virologic suppression in SHIV-SF162P3-infected rhesus macaques has previously been determined to be 1 μg/mL [13] and 22 weeks following the last antibody infusion, serum levels of PGT121 and GS-9721 had declined to below that threshold in all animals. In the bispecific+VES dosed animals, extensive anti-drug antibodies (ADAs) developed during the planned dosing period. Due to loss of exposure dosing was prematurely halted in three animals after the fourth, fifth and sixth dose of the bispecific PGT121/anti-CD3 antibody out of caution of immune hypersensitivities. Prior to the emergence of ADA, antibody serum concentrations had reached between 70 and 180 μg/mL 30 minutes post dosing and declined to between 1.7 and 11 μg/mL two weeks post dosing. Two weeks following the last bispecific PGT121/anti-CD3 dose, all animals had serum concentrations below 1 μg/mL except one animal with a serum concentration of 2.3 μg/mL.

To avoid immune hypersensitivities caused by the dosing of human antibodies in primates the emergence of ADA was evaluated during the antibody dosing period (weeks -4 to weeks 22) for animals in group 2–4 (Fig 2B) and not following antibody washout. In the PGT121+VES treatment group, none of the animals showed evidence of ADA development during the dosing period and no evidence in the post-washout PK profiles. One animal exhibited a pre-existing signal which resolved following initiation of treatment. During the dosing period of GS-9721+VES, one animal showed a high level of ADA following the last antibody dose with a corresponding impact on the PK. Following the last dose at least two additional animals exhibited evidence of ADA, characterized by an aberrant decline in exposure relative to other animals in the group. In contrast, in the bispecific+VES group, all but one animal had evidence ADA by the end of dosing despite the attempt to induce tolerance. Preliminary experiments demonstrated that two doses of a bispecific PGT121/anti-CD3KO could induce at least partial tolerance to two subsequent doses of PGT121/anti-CD3, which had showed 100% ADA after one to two doses. In this study no ADA was induced by two doses of the PGT121/anti-CD3KO and pre-treatment significantly reduced the development of ADA at least through the first five doses of the active PGT121/anti-CD3 bispecific, where only 2 of 9 animals exhibited evidence of ADA. However, the tolerance induced was not robust. Ultimately, five animals showed a high level of ADA and significant impact on PK, three animals showed low level of ADA with a minor impact on PK and only one animal did not show any sign of ADA during dosing or washout PK (Fig 2).

### VES pharmacodynamics

VES leads to innate immune activation through TLR7 activation with increases of specific cytokines/chemokines as well as immune cell activation in both primates and humans [11,24,25]. To assess the pharmacodynamic effects of VES in this study, plasma samples prior to VES dosing and 1 day following dosing were analyzed by Luminex for a set of cytokines and chemokines. As previously described, robust induction (P<0.05 for all measurement, one-way ANOVA test with Dunnett multiple-comparison correction compared with sham group) in plasma levels of IL-1RA, MCP-1, MIG, and I-TAC was observed on the day following administration (S3 Fig). The pharmacodynamic effect of VES was also assessed by immune cell activation using multiparameter flow cytometry where higher CD4+ T cell activation was observed by CD69 expression on the day following VES administration (S4 Fig). Plasma SHIV RNA loads were monitored following each VES dosing, and no virus blips were detected for any of the animals.

### Cellular immune responses to SHIV

Cellular immune responses to SHIV were monitored by Gag-, Env-, and Pol-specific CD8+ T cell responses in PBMCs pre (week -7/-8), during (week 0 and week 8), and post (week 32) antibody/VES treatments. Most animals showed low levels of Gag-, Env-, and Pol-specific cellular immune responses at the analyzed time points presumably due to the lack of antigen stimulation during the 2.5 years of ART suppression (S5 Fig).

### Viral DNA

**Fig 3:**
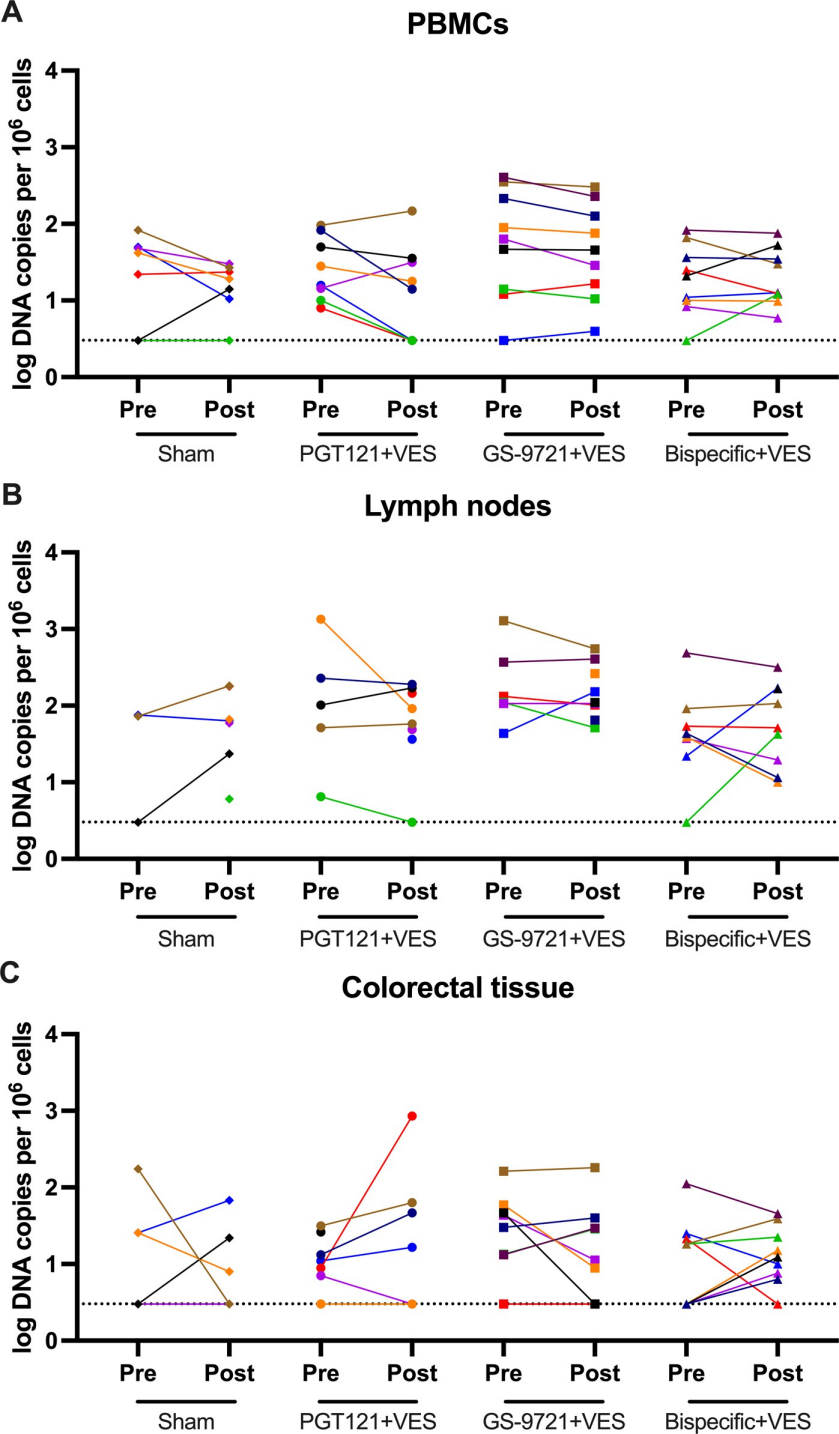
Viral DNA before discontinuation of ART. Total viral DNA in (A) PBMCs, (B) lymph nodes and (C) colorectal tissue were determined by qPCR pre (week -8) and post antibody/VES treatment (week 32). P values calculated using two-sided Mann-Whitney tests. ns, not significant (P>0.05). Dotted lines indicate limit of detection, values on the line were below limit of detection. Individual symbol and color coding as indicated in S1 Table.

Viral DNA was investigated pre (week -8) and post (week 32) antibody/VES treatments in PBMCs, lymph nodes and colorectal tissue using a quantitative PCR assay specific for total SHIV DNA. Viral DNA was detected in most animals for all tissues analyzed, however, no systematic change in viral DNA was observed post treatment for any of the groups (Fig 3A, 3B, and 3C). Viral DNA levels were, in general, higher in lymph nodes than in PBMCs and colorectal tissue, consistent with our observations in the acute ART treated model [11]. No differences in viral DNA (week -8) prior to ART discontinuation was observed for the groups for any of the tissues (S6 Fig).

### Viral rebound following ART discontinuation

**Fig 4:**
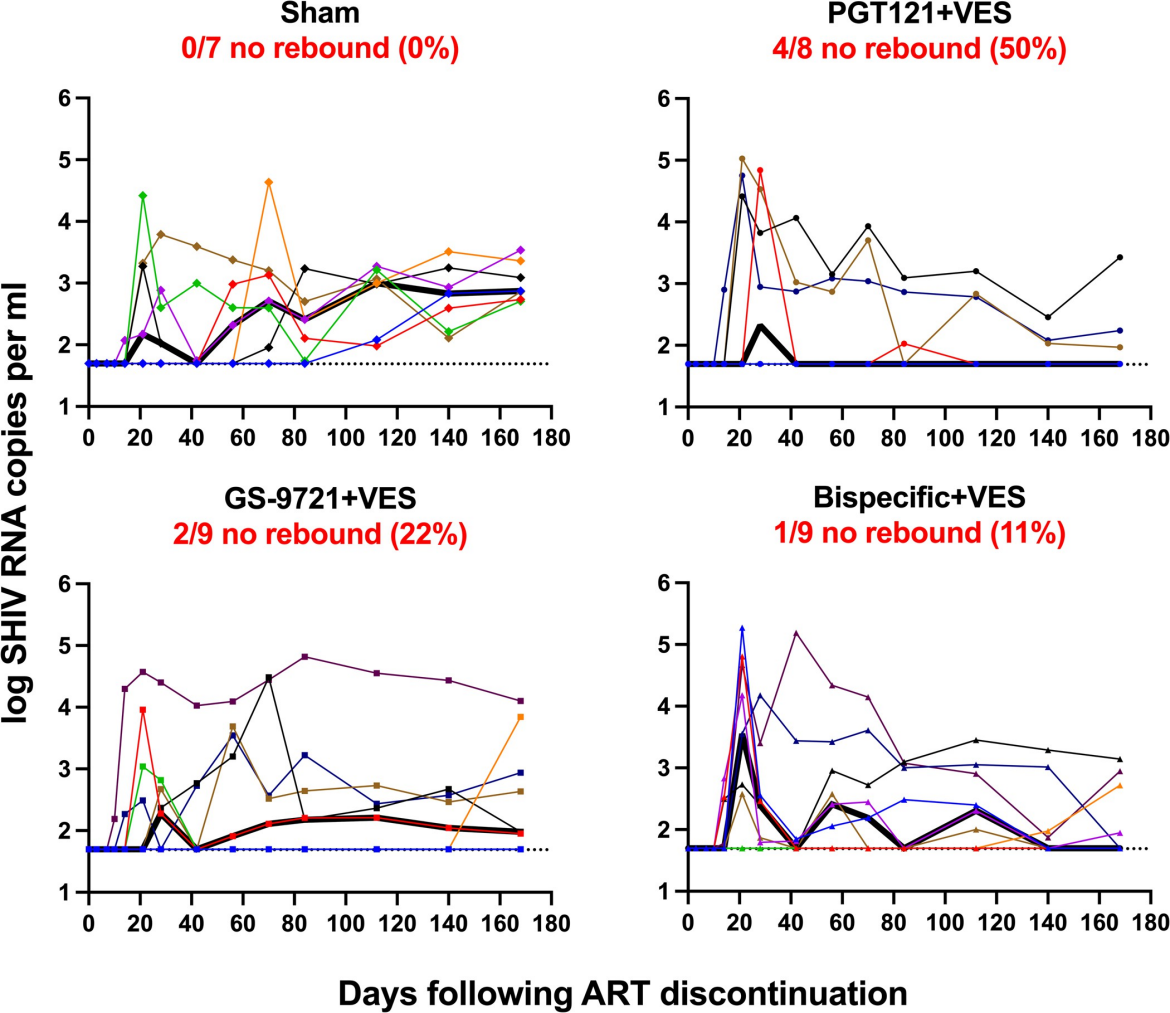
Viral loads following ART discontinuation. Plasma viral load for 168 days following ART discontinuation. Numbers and percentages of animals that did not show viral rebound are shown. In the PGT121+VES group 4 of 8 animals did not rebound (P = 0.05, Fisher’s exact test compared with sham group). In the GS-9721+VES group, 2 of 9 animals did not rebound (P = 0.30, Fisher’s exact test compared with sham group). In the bispecific+VES group, 1 of 9 animals did not rebound (P = 0.56, Fisher’s exact test compared with sham group). Dotted lines indicate limit of detection (1.7 log RNA copies per ml), values on the line were below limit of detection. Thick black lines indicate median viral load. Individual symbol and color coding as indicated in S1 Table.

At week 42 (24 weeks after the final antibody dosing in the PGT121+VES and GS-9721+VES groups to allow antibody levels to decline below therapeutic levels) ART was discontinued and animals were monitored for 24 weeks (168 days) to assess for viral rebound (Fig 4). In the sham group, 7 of 7 animals rebounded and remained viremic in the 24 weeks monitoring. In contrast, in the PGT121+VES group, 4 of 8 animals did not rebound (P = 0.05, Fisher’s exact test compared with sham group) and one animal that initially rebounded re-suppressed plasma viremia. In the GS-9721+VES group, 2 of 9 animals did not rebound (P = 0.30, Fisher’s exact test compared with sham group) and one animal that initially rebounded re-suppressed. In the bispecific+VES group, 1 of 9 animals did not rebound (P = 0.56, Fisher’s exact test compared with sham group) and three animals that initially rebounded re-suppressed.

**Fig 5:**
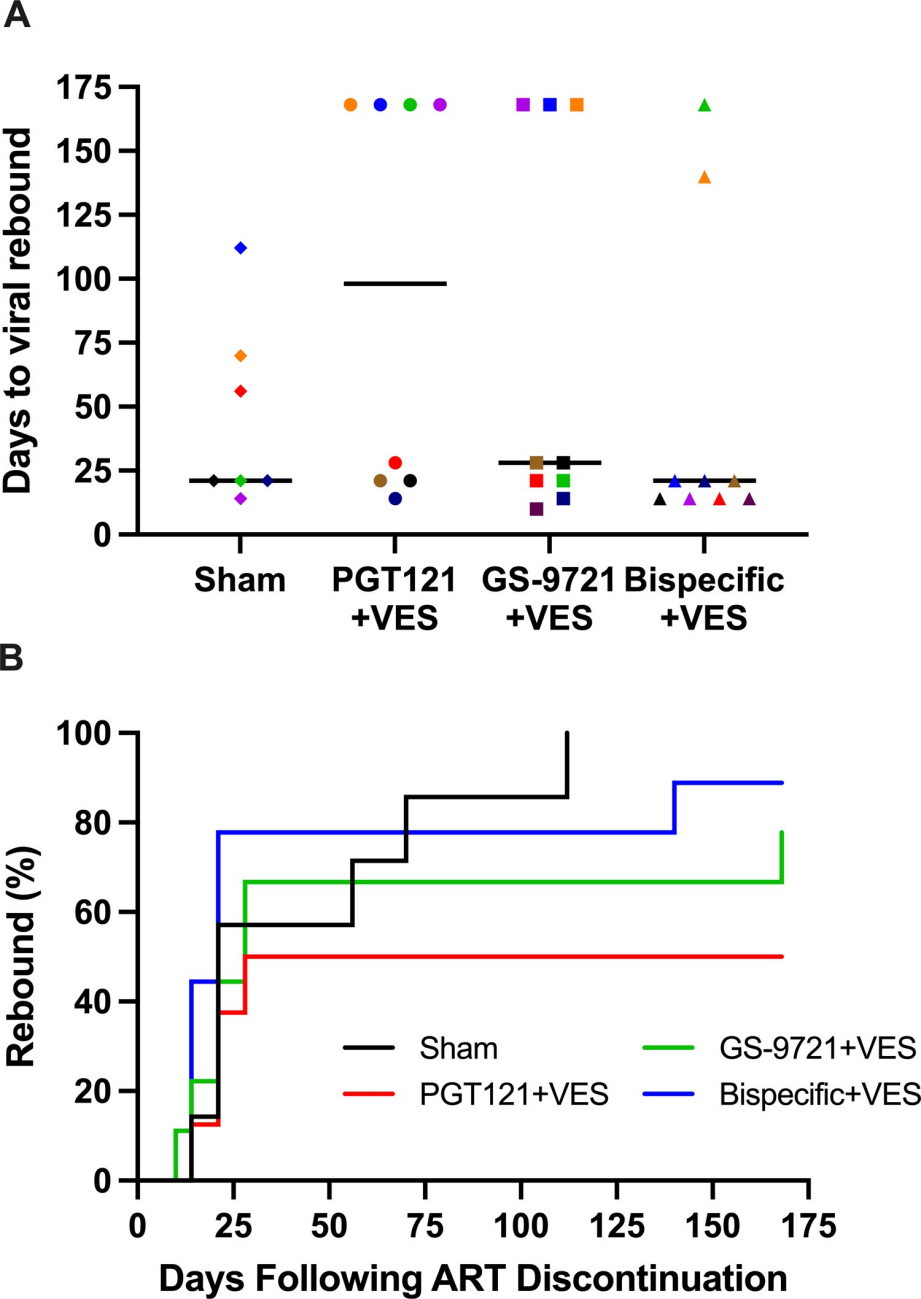
Analysis of viral loads following ART discontinuation. (A) Days to viral rebound per group. Black lines indicate median number of days for viral rebound load. Individual symbol and color coding as indicated in S1 Table. (B) Viral rebound analysis by Kaplan–Meier plot showing the percentage of rebounding animals per group as a function of time.

The median time to viral rebound in the sham group was 21 days whereas in the PGT121+VES group, it was 98 days when animals without rebound were analyzed as day 168 (Fig 5A and 5B). Interestingly, when analyzing only the animals that rebounded, the median time in the PGT121+VES group was 21 days, which was the same as in the sham group, indicating no effect of the antibody/VES treatment in those animals. The GS-9721+VES and bispecific+VES groups were comparable to the sham group with median time to viral rebound at 28 days and 21 days, respectively (Fig 5A and 5B). In the animals that rebounded, viral peak and set point were comparable across groups (S7 Fig) but in general were lower than observed with SIV. Most animals showed an increase in their cellular immune response to SHIV following ART discontinuation (S5, S8A, and S8B Figs). Comparable immune responses were observed for animals that rebounded versus animals that did not rebound at week 32 (prior to ART discontinuation) and week 66 (after ART discontinuation) (S8B Fig) as well as for animals that rebounded versus animals that rebounded and resuppressed at week 32 (prior to ART discontinuation) and week 66 (after ART discontinuation)(S8C Fig).

**Fig 6:**
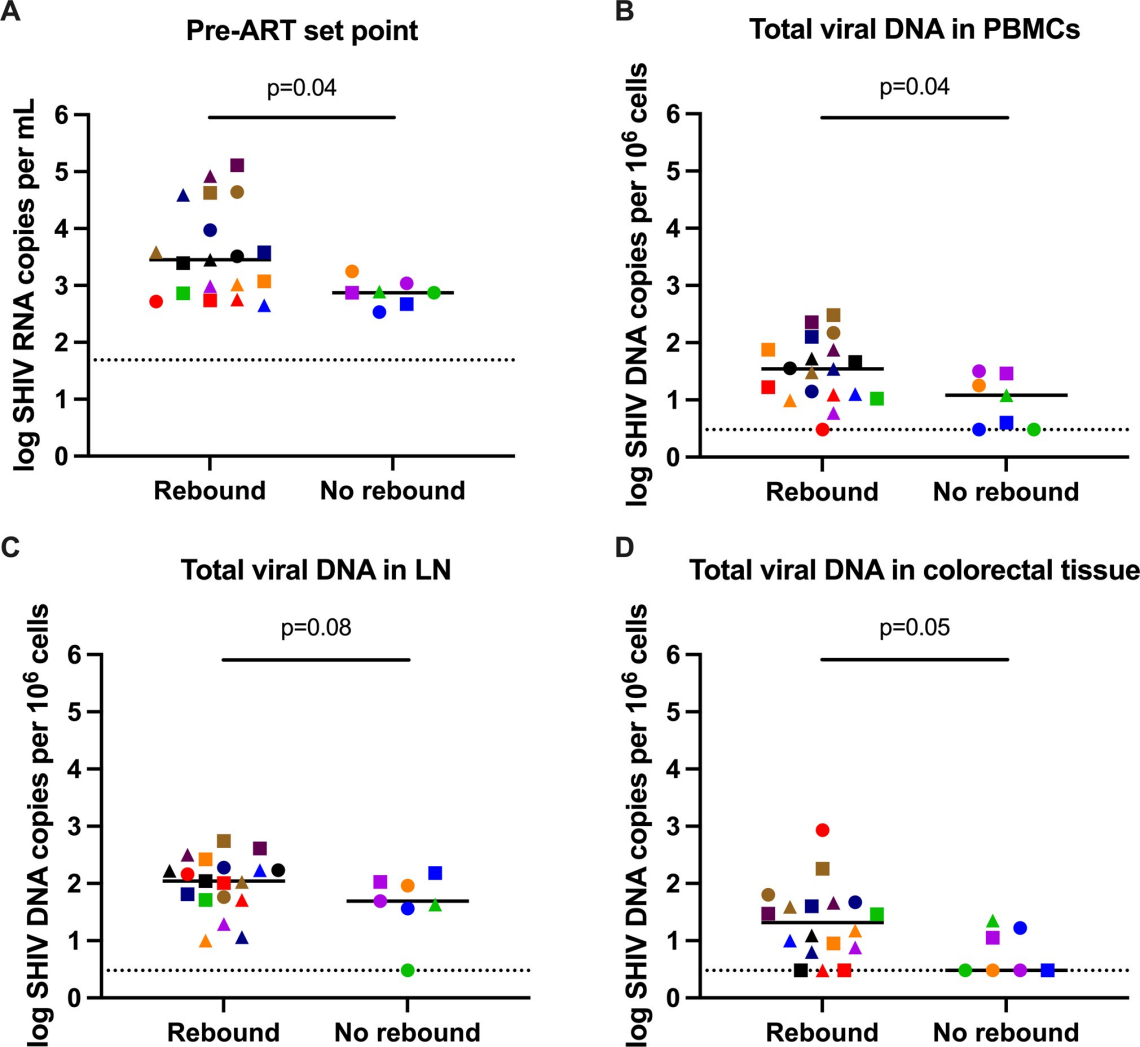
Analysis of viral rebound. Comparison of (A) pre-ART set point viral load, (B) total viral DNA in PBMCs, (C) total viral DNA in lymph nodes and (D) total viral DNA in colorectal tissue at week 32 for animals that rebounded and animals that did not rebound following ART discontinuation. Dotted lines indicate limit of detection, values on the line were below limit of detection. Black lines indicate median values. Individual symbol and color coding as indicated in S1 Table.

Comparing pre-ART plasma viral set points showed that animals that did not rebound following ART discontinuation had lower set points compared to those that did rebound (P = 0.041, Mann–Whitney test) (Figs 6A and S9A). In addition, the viral reservoir at week 32 (prior to ART discontinuation) based on total SHIV DNA in PBMCs was smaller in the animals that did not rebound compared to the animals that did rebound (P = 0.04, Mann–Whitney test) and trending toward smaller in lymph nodes (P = 0.08, Mann–Whitney test) and colorectal tissue (P = 0.05, Mann–Whitney test) (Figs 6B, 6C, 6D, S9B, S9C, and S9D). No difference was observed in antibody exposure between animals that rebounded and animals that did not rebound (S10 Fig).

### CD8+ cell depletion

**Fig 7:**
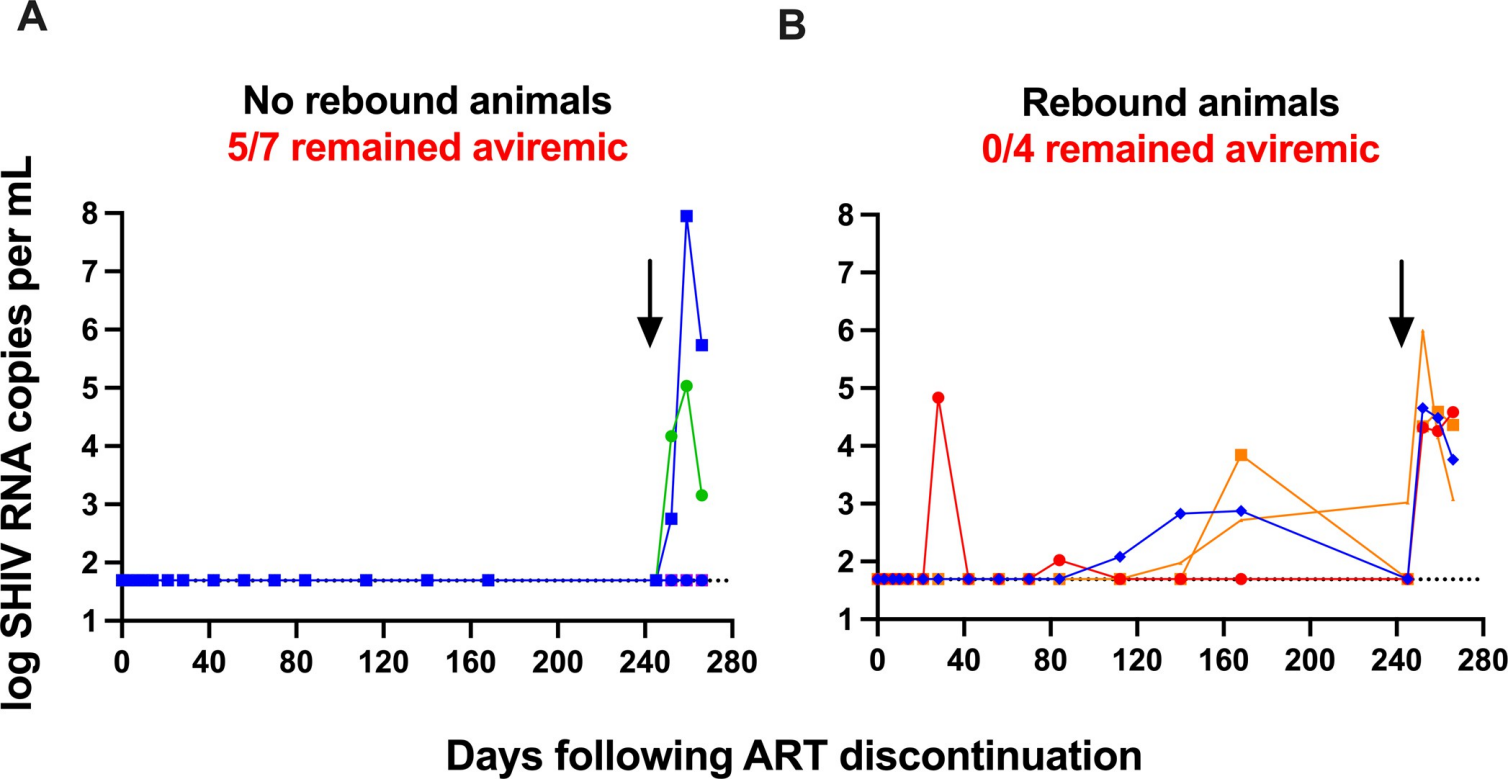
CD8+ cell depletion. Plasma viral loads before and after CD8+ cell depletion in (A) animals with no viral rebound (n = 7) and (B) animals with viral rebound (n = 4) following ART discontinuation. Numbers of animals that remained aviremic and total number of animals are shown. Arrows indicate CD8+ cell depletion on day 245 (week 77) after ART discontinuation. Dotted lines indicate limit of detection, values on the line were below limit of detection. Individual symbol and color coding as indicated in S1 Table.

To explore whether low level residual replication competent virus was present in the 7 animals (4 from the PGT121+VES group, 2 from the GS-9721+VES group, and 1 from the bispecific- +VES group) that did not rebound following ART discontinuation, we performed a CD8+ T and NK cell depletion at week 77. A single intravenous infusion of an anti-CD8α CDR-grafted rhesus IgG1 antibody resulted in efficient CD8+ T cell depletion in PBMCs (S11 Fig). Importantly, of the 7 animals that did not rebound after ART discontinuation, 5 remained aviremic following the CD8+ T and NK cell depletion demonstrating that virologic control was not mediated by CD8+ T or NK cells in these animals (Fig 7A). In contrast, CD8+ T and NK cell depletion in 4 animals that rebounded (all low level viremia) following ART discontinuation resulted in plasma virus spikes in all animals, including 3 animals that were aviremic at the time of depletion demonstrating that in these animals, viral control was CD8+ T or NK cell mediated (Fig 7B).

## Discussion

In this study, we demonstrate that a HIV bNAb formatted in three immune cell engager formats, together with a TLR7 agonist can partially prevent viral rebound following discontinuation of ART in SHIV-infected rhesus macaques that initiated ART 1 year into chronic infection. To the best of our knowledge, no similar study has been performed in nonhuman primates in animals that started ART deep into chronic infection. Importantly, in most of the animals that did not rebound following the ART discontinuation, CD8+ cell depletion did not result in plasma viral rebound, suggesting that the lack of rebound was not dependent on CD8+ T or NK cells. This proof-of-concept study in chronically SHIV-infected rhesus monkeys supports evaluation of these strategies in humans.

Our data build upon previous observations that bNAbs together with TLR7 activation can delay or prevent viral rebound following ART discontinuation presumably by reducing or eliminating the replication-competent viral reservoir [11,12]. In this study, animals progressed into chronic infection before ART was initiated to allow for the formation of a viral reservoir that more closely resembles the HIV-1 reservoir in infected people. Our results in this study of chronically infected monkeys were comparable to what we observed in the acute ART model [11]. As in our previous study, virologic control after stopping ART was not due to the direct antiviral effect of the bNAb and VES regimen as ART was released only after antibody concentrations had waned to below therapeutic levels. We cannot rule out that the animals without viral rebound potentially still contain residual replication-competent virus despite the long ATI period and anti-CD8 depletion as viral rebound can occur in humans following long periods off ART [26,27]. However, we demonstrated that CD8+ cell depletion in the monkeys that rebounded after stopping ART (and then spontaneously resuppressed) led to viral rebound, indicating that CD8+ T and NK cell depletion is capable of exposing replication competent virus in animals with undetectable plasma viremia. Conversely, we also observed monkeys that did not rebound after stopping ART and subsequently treated with the anti-CD8 antibody did not rebound indicating that these animals may be depleted of replication-competent virus.

Virologic analyses showed that animals that did not rebound following ART discontinuation had lower pre-ART plasma set points and smaller viral reservoirs than animals that did rebound, however, specific treatment induced differences were not identified. The viral reservoir was evaluated using qPCR of the total SHIV DNA, which does not distinguish between defective and replication-competent virus. We also attempted to measure the replication-competent virus reservoir using the intact proviral DNA assay (IPDA) [28]. Unfortunately, using a SHIV-modified IPDA, we found that levels of intact virus were too low across the animals tested, including the sham controls, likely as a result of the long period of ART suppression coupled with poor recovery yields of the cryopreserved PBMCs. Further research is required to define the mechanism of action of these interventions.

High level of ADA was observed for the bispecific-PGT121/antiCD3 antibody which limited our ability to compare the three antibody formats used in this study. Nevertheless, whereas all animals from the sham group rebounded following ART discontinuation, a single animal from the bispecific+VES group did not rebound (despite exhibiting evidence of ADA following the 5^th^ dose). In addition, this animal remained aviremic following the CD8+/NK cell depletion suggesting that the combination of this bispecific antibody with TLR7 activation may be capable of reducing replication competent reservoir. This result supports that further investigation of T-cell engaging antibodies for HIV cure is warranted. For the effector enhanced antibody GS-9721, we did not see any increase in efficacy compared to the wildtype PGT121 IgG antibody. As GS-9721 did not appear to be markedly influenced by ADA, our data suggests that effector enhancement via the DEAL mutations in the Fc domain (S239D, I332E, G236A, A330L) may not be essential in the SHIV model. How this observation may translate to cure studies in PLWH is unclear, and may require additional studies, as the DEAL mutation was engineered to optimize human FcgRIIIA, IIA and IIB binding properties, not all of which may translate equivalently to NHPs [16,17,18]. High level of ADA was also observed in a recent study using the bNAbs PGT121 and N6 paired with a TLR7 agonist in SHIV-1157ipd3N4 infected rhesus macaques [12]. In that study, all animals rebounded following treatment interruption although the animals dosed with the bNAbs and TLR7 agonist did show a delay in viral rebound (relative to control animals), despite having substantial ADA which reduced antibody exposures. The observed ADA in that study limited the number of antibody doses of the two bNAbs which presumably restricted the overall antiviral effect.

In summary, our data shows that a bNAb combined with innate immune stimulation can partially prevent viral rebound following discontinuation of ART in animals initiating ART during chronic infection. However, important virologic and immunologic differences exist between the macaque model and humans. Nevertheless, our findings encourage clinical studies to evaluate such strategies to achieve HIV remission in humans.

## Materials and methods

### Ethics statement

33 Indian-origin adult rhesus macaques were housed at Alpha Genesis Inc., Yemassee, SC. All animal studies complied with all relevant ethical regulations and were approved by the Alpha Genesis Institutional Animal Care and Use Committee (IACUC).

### Animals and study design

Animals were infected intrarectally with a single 500 TCID_50_ dose of a rhesus PBMC derived SHIV-SF162P3 stock [22]. Antiretroviral therapy, 5.1 mg/mL tenofovir disoproxil fumarate (TDF), 40 mg/mL emtricitabine (FTC), and 2.5 mg/mL dolutegravir (DTG) in water containing 15% (v/v) kleptose pH 4.2, was initiated 1 year after infection and administered subcutaneous once daily at 1 mL/kg body weight. 2.5 years after ART initiation, animals were stratified by viral set points (S1 Table) before the following interventions were starting: Sham (Group 1, n = 7), PGT121 and VES (Group 2, n = 8), GS-9721 and VES (Group 3, n = 9) or bispecific-PGT121/antiCD3 (GS-968989) and VES (Group 4, n = 9) (Fig 1). In Group 2 and 3, animals received 10 biweekly doses of antibody (intravenous infusions of 10 mg/Kg) and VES (oral administrations of 0.15 mg/Kg) with the first administration of VES on the day of the third antibody administration. Animals in Group 4 received first 2 biweekly doses of bispecific-PGT121 with an antiCD3 knock out arm (GS-969133) (intravenous infusions of 5 mg/Kg) followed by 10 biweekly doses of bispecific-PGT121/antiCD3 (intravenous infusions of 5 mg/Kg) and VES (oral administrations of 0.15 mg/Kg) with the first administration of VES on the day of first bispecific- PGT121/antiCD3 administration. PGT121 was produced at Catalent Biopharma (Madison, WI), GS-9721, bispecific-PGT121/antiCD3 (GS-968989), bispecific-PGT121 with an antiCD3 knock out arm (GS-969133) and VES were produced at Gilead Sciences (Foster City, CA). ART was discontinued at 24 weeks after the final combined antibody/VES doses and viral rebound was monitored for an additional 24 weeks. At week 77, CD8+ cell depletion was performed in a subset of animals.

### CD8 depletion studies

Animals received a single intravenous infusion of 50 mg/kg of the anti-CD8α CDR-grafted rhesus IgG1 antibody MT807R1 (Keith Reimann, MassBiologics, Mattapan, MA) to deplete CD8+ cells. CD8+ T cell counts and viral loads were assessed weekly following anti-CD8 infusion.

### Pharmacodynamics

Cytokine and chemokine levels were determined by the Monkey Cytokine Magnetic 29-Plex Panel (Life Technologies Corporation, Carlsbad, CA) for the Luminex platform following the manufacturer’s instructions. Cell activation was assessed by multiparameter flow cytometry by % CD69 expression on the CD3+/CD4+ population for CD4+ T cells as previously described [11].

### Pharmacokinetics

All antibodies were detected in rhesus monkey serum using Meso Scale Discovery (MSD) based electrochemiluminescence (ECL) methods of sufficient sensitivity and specificity. Each MSD-ECL assay utilized standard MSD plates coated with gp120 (Bal) HIV-1 envelope antigen (IT-001-002p, Immune Technology, New York, NY). Briefly, serum from individual animals, standards, and controls were captured onto gp120 coated plates. Following thorough washing to remove unbound antibodies, PGT121 and GS-9721 were detected with goat anti-human IgG1 biotin conjugated secondary antibody (Southern Biotech, Birmingham, AL) and ruthenylated conjugate streptavidin. GS-968989 and GS-969133 were detected using an in-house developed ruthenylated conjugated anti-idiotype antibody against the anti-CD3 variable domain. After incubations and washings, a read buffer was added, and plates are analyzed with an MSD plate reader. The ECL signal produced is proportional to the amount of antibody bound to the plate. The concentrations of antibodies in controls and samples are back calculated with a standard curve established with each antibody using a 4-parameter logistic curve fit. PK was monitored to below 1 μg/mL (except in one of the GS-9721 treated animals) as this is the threshold for PGT121-mediated virologic suppression in SHIV-SF162P3-infected rhesus macaques. The final number of measurements was dependent on sample available.

### Anti-drug antibody (ADA) assays

Specific MSD-ECL assays were developed for detection of ADA in serum from PGT121, GS-9721, GS-968989 and GS-969133 treated rhesus monkeys. All ADA assays were bridging format MSD-ECL assays that utilized the respective biotin labeled antibody as an ADA capture reagent on streptavidin-coated MSD microtiter plates, incubated with monkey serum and the respective ruthenylated antibody as a detection reagent. Animals were considered ADA positive if a relative light signal (RLU) greater than 2-fold pre-dose was observed. The strength of the ADA response was proportional to the magnitude of the RLU signal.

### Cellular immune assays

SIV-specific cellular immune responses were assessed using ELISPOT as previously described [23]. Briefly, ELISPOT plates were coated with an anti-IFN-γ monoclonal antibody and PBMCs and SIV Gag, SIV Pol or HIV Env peptide pools were added to the plates and incubated for 1 day before processing and readout.

### Viral RNA and DNA assays

Viral RNA was isolated from cell-free plasma and quantified essentially as previously described [29]. Total SHIV DNA was quantified from isolated total cellular DNA by qPCR using SIVmac239 specific primers as previously described [29].

### Statistical analyses

Analyses were performed using GraphPad Prism 8 (GraphPad Software). Comparisons of groups were performed using two-sided Mann–Whitney tests without Bonferroni adjustments. Comparisons of number of animals with/without rebound for each treatment group vs. sham were performed using one-sided Fisher’s exact tests. Comparisons of VES induction vs sham group were performed using one-way ANOVA test with Dunnett multiple-comparison correction. Comparisons of Viral DNA across groups were performed using the Kruskal-Wallis test. No statistical methods were used to predetermine sample size.

## Supporting information

S1 Table Viral set points. (XLSX)

S1 Fig Schematic representation of the PGT121 antibody formats. PGT121 is a wildtype human IgG1 antibody. GS-9721 (Thomsen et al., Conference on Retroviruses and Opportunistic Infections 2019) is an engineered version of PGT121 that contains the S239D, I332E, G236A, A330L, M428L and N434S point mutations in the Fc domain for enhanced Fc-mediated effector functions and half-life through higher binding affinities for activating Fcγ receptors (FcγRs) and the neonatal Fc receptor (FcRn). Bispecific PGT121/anti-CD3 is generated in the DuoBody platform and contains a PGT121 Fv, an anti-CD3 Fv (Van Den Brink et al. Humanized or Chimeric CD3 Antibodies. United States Patent US 10,465,006 B2. United States Patent and Trademark Office. 5 Nov. 2019) that binds both human and NHP CD3 epsilon, and a rhesus Fc domain. Bispecific PGT121/anti-CD3 is engineered to have reduced Fc-mediated effector functions (Labrijn et al. Inert Format. United States Patent US 10,590,206 B2. United States Patent and Trademark Office. 17 Mar. 2020). PGT121 Fv* indicates the use of PGT121 Fv variants with improved manufacturing properties and nearly identical functional parameters. Fv# indicates that the anti-CD3 Fv in the Bispecific PGT121/anti-CD3 antibody was engineered in a CD3 binding and CD3 nonbinding version.
(TIFF)

S2 Fig Viral loads following ART initiation. Plasma viral load for 116 weeks following ART initiation. Second measurement is on day 1 following ART initiation. Dotted lines indicate limit of detection (1.7 log RNA copies per ml), values on the line were below limit of detection. Individual symbol and color coding as indicated in S1 Table.
(TIFF)

S3 Fig Plasma cytokine and chemokine induction following VES administration before ART discontinuation. IL-1RA, MCP-1, MIG and I-TAC are shown on day 1 following VES administration. Data combined from all VES administrations with pre-dose levels subtracted. Red lines indicate median values. P<0.05 for all measurement, one-way ANOVA test with Dunnett multiple-comparison correction compared with sham group. Dotted lines mark value 0.
(TIFF)

S4 Fig Immune cell activation following VES administration before ART discontinuation. Activation of CD4+ T cells was assessed by CD69 expression on days 0 and 1 following VES administration. Representative data are shown following the first VES dose. Red lines indicate median values. P values calculated using two-sided Mann-Whitney tests. ns, not significant (P>0.05)
(TIFF)

S5 Fig ELISPOT responses in PBMCs before ART discontinuation. Gag-, Env-, and Pol-specific IFNγ responses are shown for each animal at week -7/-8, week 0, week 8 and week 32 as spot-forming cells (SFCs) per million PBMCs. Red lines indicate median values.
(TIFF)

S6 Fig Viral DNA before discontinuation of ART. Total viral DNA in PBMCs, lymph nodes and colorectal tissue were determined by qPCR pre treatment (week -8). No difference in viral DNA between the groups for any of the tissues (P = 0.40, P = 0.12 and P = 0.44 in PBMCs, lymph nodes and colorectal tissue, respectively, Kruskal-Wallis test). Dotted lines indicate limit of detection, values on the line were below limit of detection. Red lines indicate median values.
(TIFF)

S7 Fig Viral peak and set point following ART discontinuation. (a) Peak and (b) set point viral loads following ART discontinuation are shown for each animal. Red lines indicate median values.
(TIFF)

S8 Fig ELISPOT responses in PBMCs before (week 32) and after (week 66) ART discontinuation. (A) Gag-, Env-, and Pol-specific IFNγ responses are shown for each animal at week 66 as spot-forming cells (SFCs) per million PBMCs. (B) Combined Gag-, Env-, and Pol-specific IFNγ responses are shown for all animal, for rebounding animals and for no rebound animals at week 32 and week 66 as spot-forming cells (SFCs) per million PBMCs. (C) Combined Gag-, Env-, and Pol-specific IFNγ responses at week 32 (P = 0.56, Mann-Whitney test) and week 66 (P = 0.65, Mann-Whitney test) are shown for rebounding animals and for rebounding animals that resupress virus as spot-forming cells (SFCs) per million PBMCs. Red lines indicate median values.
(TIFF)

S9 Fig Analysis of viral rebound. Comparison of (A) pre-ART set point viral load, (B) total viral DNA in PBMCs, (C) total viral DNA in lymph nodes and (D) total viral DNA in colorectal tissue at week 32 per antibody/VES treatment group for animals that rebounded and animals that did not rebound following ART discontinuation. Dotted lines indicate limit of detection, values on the line were below limit of detection. Red lines indicate median values.
(TIFF)

S10 Fig Analysis of viral rebound and antibody exposure. Comparison of antibody exposure per antibody/VES treatment group for animals that rebounded and animals that did not rebound following ART discontinuation. Antibody exposure assessed as area under the curve (AUC). Red lines indicate median values.
(TIFF)

S11 Fig CD8+ T cell depletion efficiency. CD8+ T cells per μl peripheral blood are shown before and after CD8 depletion in animals with no viral rebound (n = 7, left) and in animals with viral rebound (n = 4, right) following ART discontinuation.
(TIFF)
