# Supplementary figures and images for: HIV envelope antibodies and TLR7 agonist partially prevent viral rebound in chronically SHIV-infected monkeys
Source: PLoS Pathog. 2022 Apr 22;18(4):e1010467. doi: 10.1371/journal.ppat.1010467 (PMC9067686; doi:10.1371/journal.ppat.1010467)

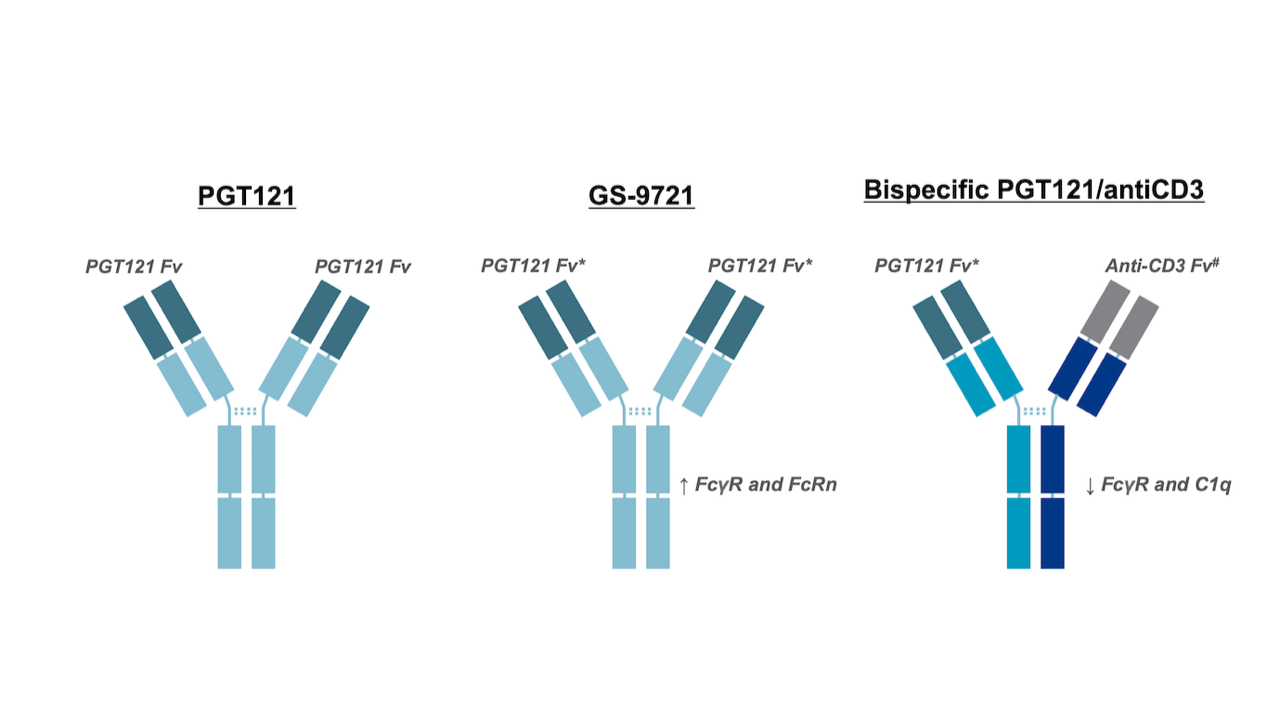

Supplement: S1 Fig — PGT121 is a wildtype human IgG1 antibody. GS-9721 (Thomsen et al., Conference on Retroviruses and Opportunistic Infections 2019) is an engineered version of PGT121 that contains the S239D, I332E, G236A, A330L, M428L and N434S point mutations in the Fc domain for enhanced Fc-mediated effector functions and half-life through higher binding affinities for activating Fcγ receptors (FcγRs) and the neonatal Fc receptor (FcRn). Bispecific PGT121/anti-CD3 is generated in the DuoBody platform and contains a PGT121 Fv, an anti-CD3 Fv (Van Den Brink et al. Humanized or Chimeric CD3 Antibodies. United States Patent US 10,465,006 B2. United States Patent and Trademark Office. 5 Nov. 2019) that binds both human and NHP CD3 epsilon, and a rhesus Fc domain. Bispecific PGT121/anti-CD3 is engineered to have reduced Fc-mediated effector functions (Labrijn et al. Inert Format. United States Patent US 10,590,206 B2. United States Patent and Trademark Office. 17 Mar. 2020). PGT121 Fv* indicates the use of PGT121 Fv variants with improved manufacturing properties and nearly identical functional parameters. Fv# indicates that the anti-CD3 Fv in the Bispecific PGT121/anti-CD3 antibody was engineered in a CD3 binding and CD3 nonbinding version. (TIFF) [file ppat.1010467.s002.tiff]

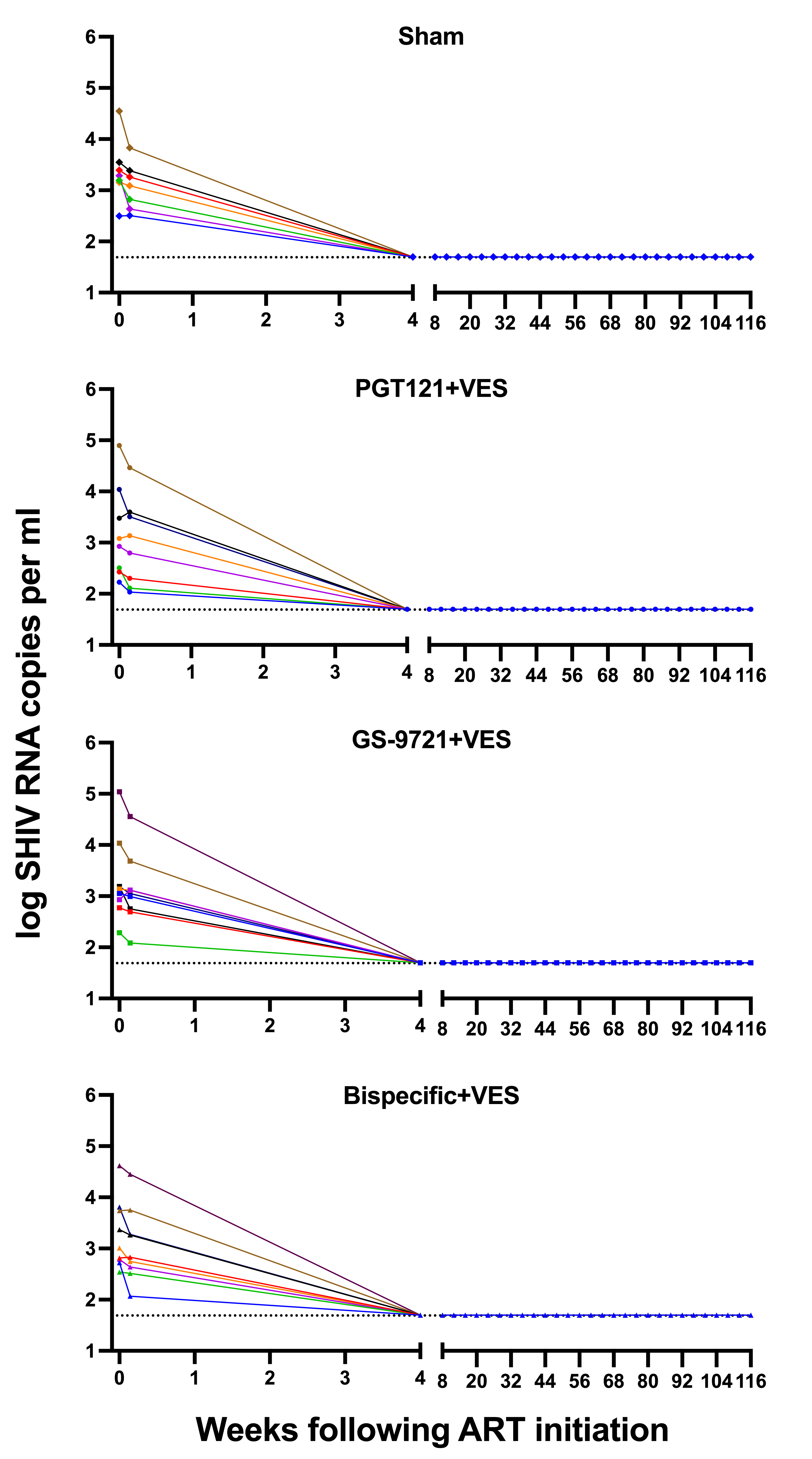

Supplement: S2 Fig — Plasma viral load for 116 weeks following ART initiation. Second measurement is on day 1 following ART initiation. Dotted lines indicate limit of detection (1.7 log RNA copies per ml), values on the line were below limit of detection. Individual symbol and color coding as indicated in S1 Table. (TIFF) [file ppat.1010467.s003.tiff]

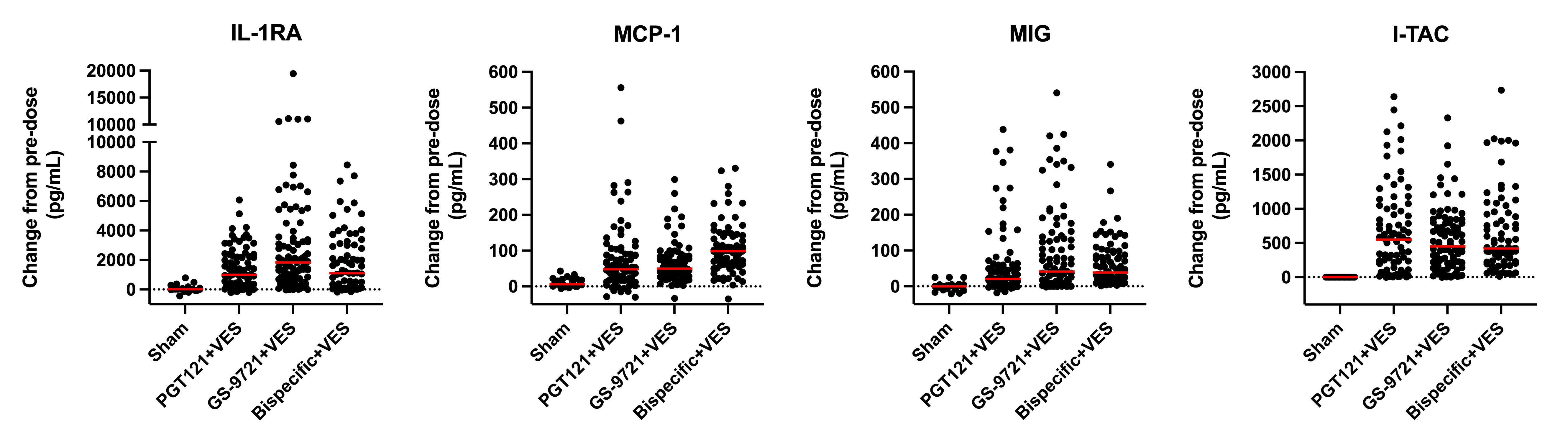

Supplement: S3 Fig — IL-1RA, MCP-1, MIG and I-TAC are shown on day 1 following VES administration. Data combined from all VES administrations with pre-dose levels subtracted. Red lines indicate median values. P<0.05 for all measurement, one-way ANOVA test with Dunnett multiple-comparison correction compared with sham group. Dotted lines mark value 0. (TIFF) [file ppat.1010467.s004.tiff]

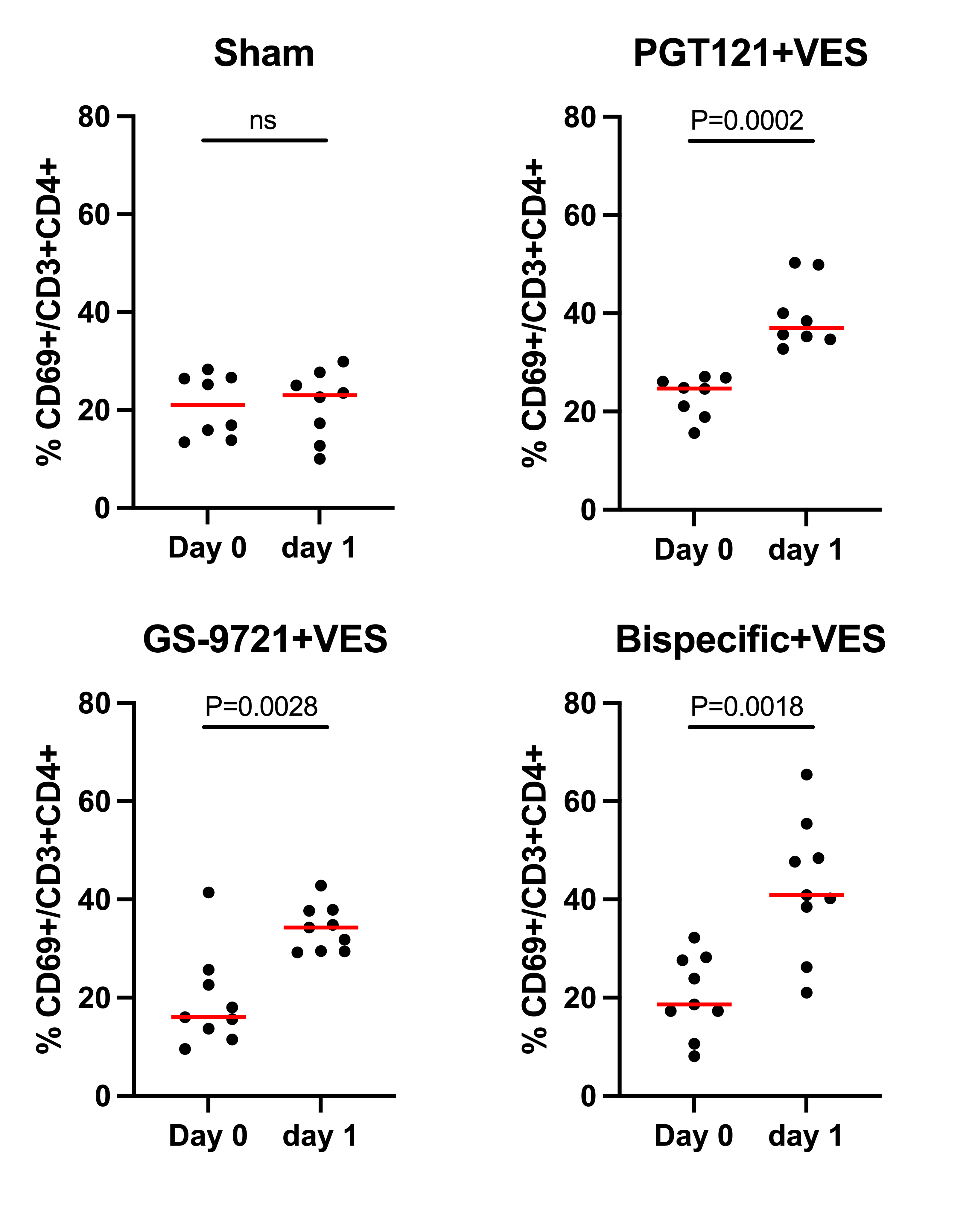

Supplement: S4 Fig — Activation of CD4+ T cells was assessed by CD69 expression on days 0 and 1 following VES administration. Representative data are shown following the first VES dose. Red lines indicate median values. P values calculated using two-sided Mann-Whitney tests. ns, not significant (P>0.05) (TIFF) [file ppat.1010467.s005.tiff]

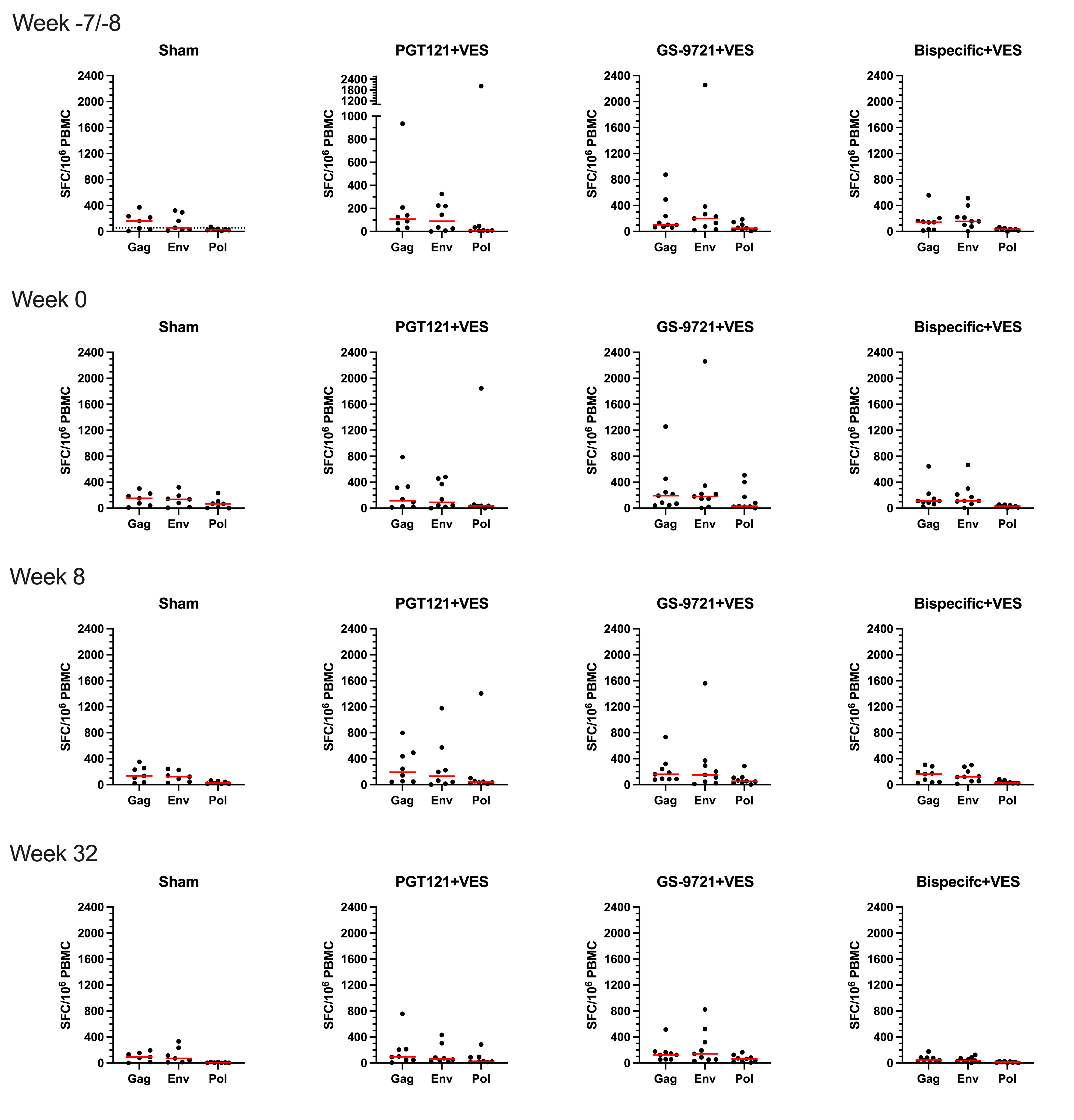

Supplement: S5 Fig — Gag-, Env-, and Pol-specific IFNγ responses are shown for each animal at week -7/-8, week 0, week 8 and week 32 as spot-forming cells (SFCs) per million PBMCs. Red lines indicate median values. (TIFF) [file ppat.1010467.s006.tiff]

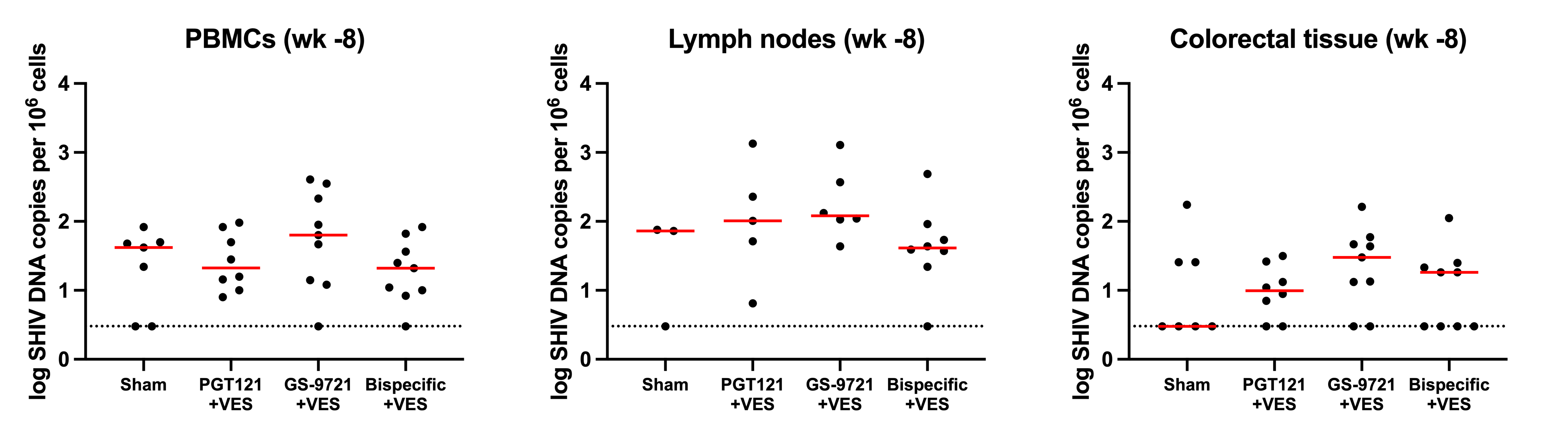

Supplement: S6 Fig — Total viral DNA in PBMCs, lymph nodes and colorectal tissue were determined by qPCR pre treatment (week -8). No difference in viral DNA between the groups for any of the tissues (P = 0.40, P = 0.12 and P = 0.44 in PBMCs, lymph nodes and colorectal tissue, respectively, Kruskal-Wallis test). Dotted lines indicate limit of detection, values on the line were below limit of detection. Red lines indicate median values. (TIFF) [file ppat.1010467.s007.tiff]

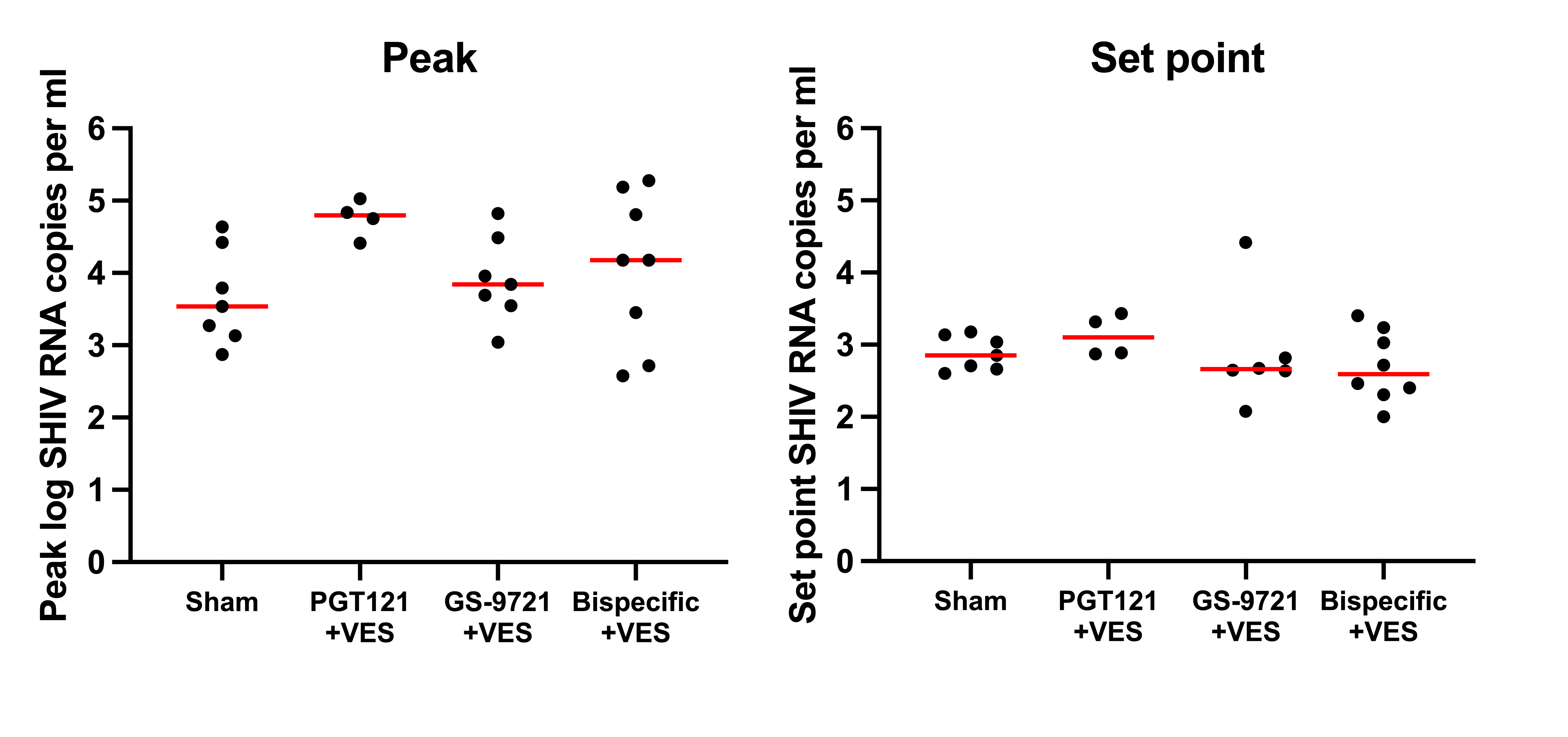

Supplement: S7 Fig — (a) Peak and (b) set point viral loads following ART discontinuation are shown for each animal. Red lines indicate median values. (TIFF) [file ppat.1010467.s008.tiff]

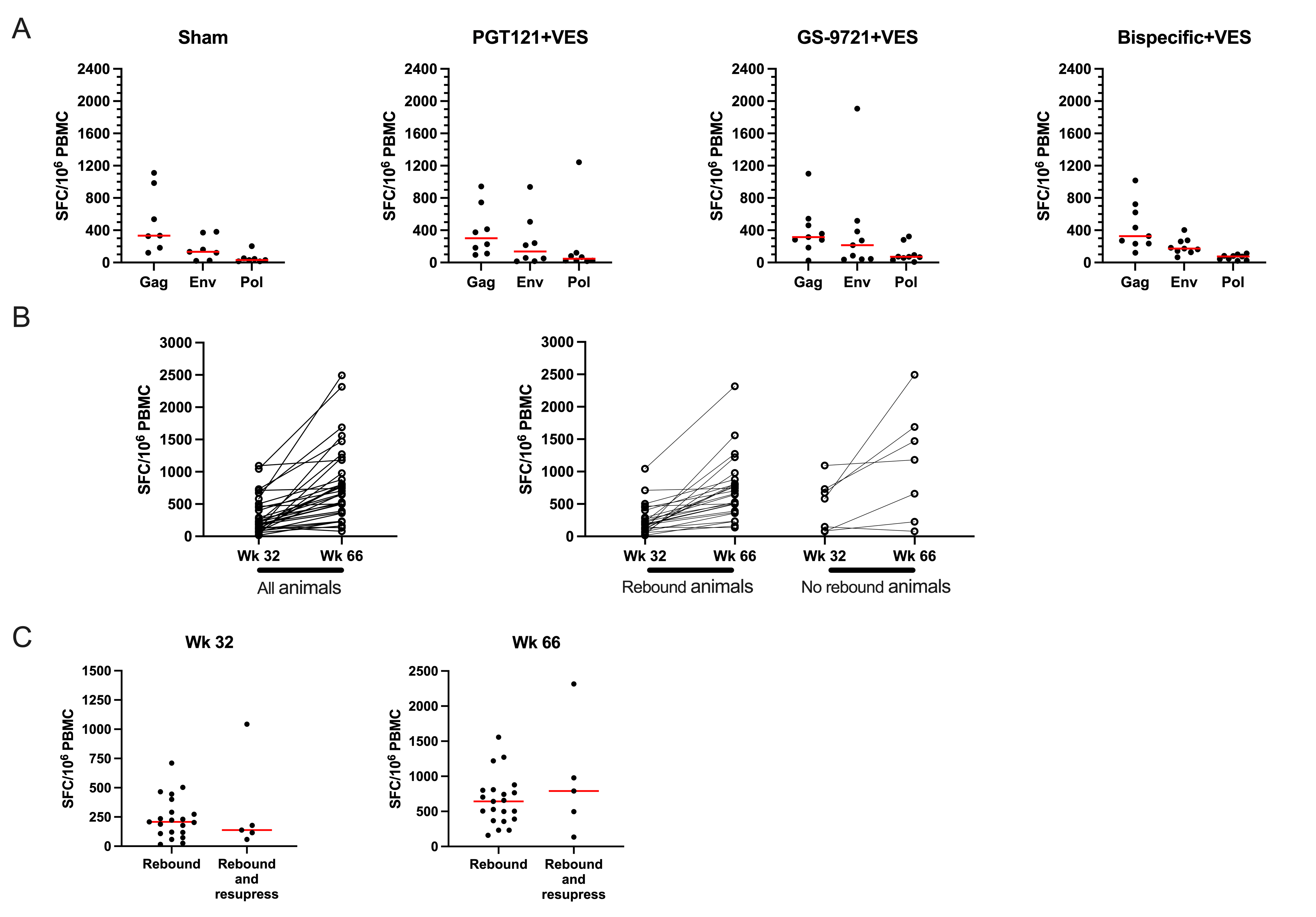

Supplement: S8 Fig — (A) Gag-, Env-, and Pol-specific IFNγ responses are shown for each animal at week 66 as spot-forming cells (SFCs) per million PBMCs. (B) Combined Gag-, Env-, and Pol-specific IFNγ responses are shown for all animal, for rebounding animals and for no rebound animals at week 32 and week 66 as spot-forming cells (SFCs) per million PBMCs. (C) Combined Gag-, Env-, and Pol-specific IFNγ responses at week 32 (P = 0.56, Mann-Whitney test) and week 66 (P = 0.65, Mann-Whitney test) are shown for rebounding animals and for rebounding animals that resupress virus as spot-forming cells (SFCs) per million PBMCs. Red lines indicate median values. (TIFF) [file ppat.1010467.s009.tiff]

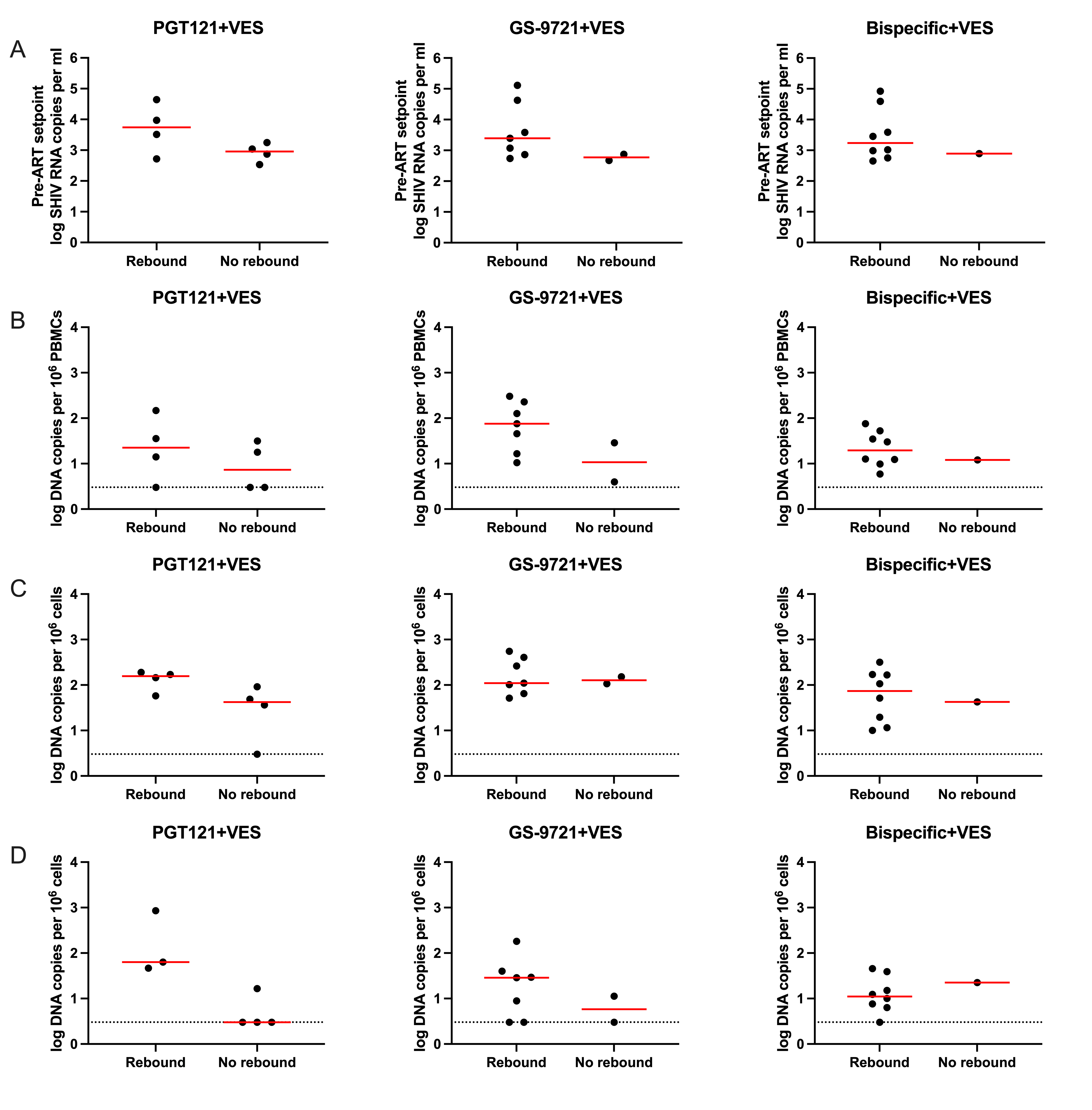

Supplement: S9 Fig — Comparison of (A) pre-ART set point viral load, (B) total viral DNA in PBMCs, (C) total viral DNA in lymph nodes and (D) total viral DNA in colorectal tissue at week 32 per antibody/VES treatment group for animals that rebounded and animals that did not rebound following ART discontinuation. Dotted lines indicate limit of detection, values on the line were below limit of detection. Red lines indicate median values. (TIFF) [file ppat.1010467.s010.tiff]

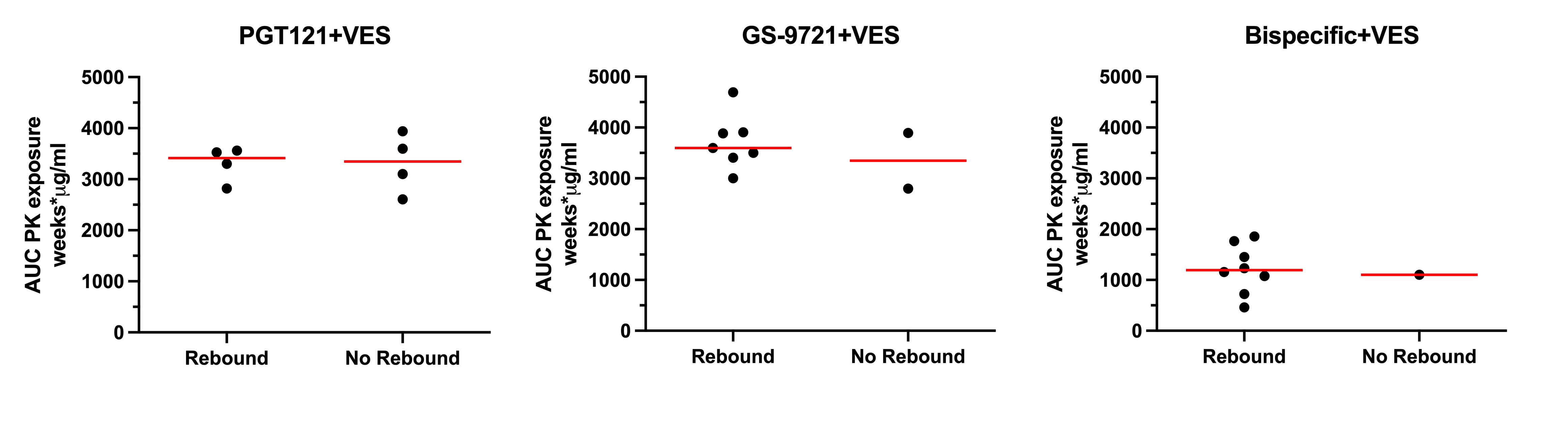

Supplement: S10 Fig — Comparison of antibody exposure per antibody/VES treatment group for animals that rebounded and animals that did not rebound following ART discontinuation. Antibody exposure assessed as area under the curve (AUC). Red lines indicate median values. (TIFF) [file ppat.1010467.s011.tiff]

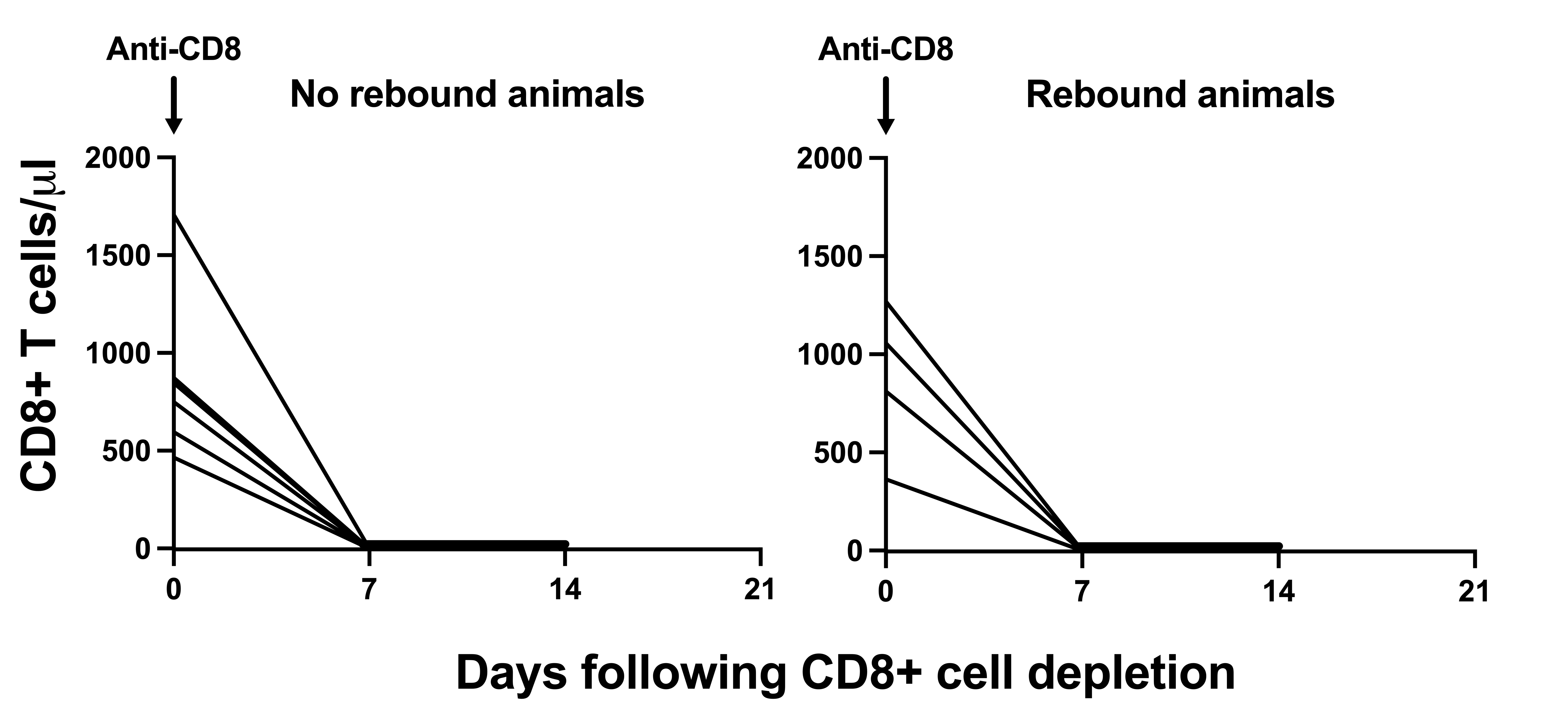

Supplement: S11 Fig — CD8+ T cells per μl peripheral blood are shown before and after CD8 depletion in animals with no viral rebound (n = 7, left) and in animals with viral rebound (n = 4, right) following ART discontinuation. (TIFF) [file ppat.1010467.s012.tiff]
